# Impact of Bacterial Etiology on Procalcitonin, C-reactive Protein and Hematological Parameters: Evaluating Mean Platelet Volume for Differentiating Gram-Negative and Gram-Positive Bacteria in Odontogenic Versus Non-odontogenic Head and Neck Abscesses

**DOI:** 10.7759/cureus.69352

**Published:** 2024-09-13

**Authors:** Gergana M Chausheva, Yanko G Yankov, Diana D Nenova

**Affiliations:** 1 Central Clinical Laboratory, University Hospital "St. Marina", Varna, BGR; 2 Department of Clinical Laboratory, Medical University "Prof. Dr. Paraskev Stoyanov", Varna, BGR; 3 Clinic of Maxillofacial Surgery, University Hospital "St. Marina", Varna, BGR; 4 Department of General and Operative Surgery, Medical University "Prof. Dr. Paraskev Stoyanov", Varna, BGR; 5 Clinic of Nephrology and Dialysis, University Hospital "St. Marina", Varna, BGR; 6 Second Department of Internal Disease, Medical University "Prof. Dr. Paraskev Stoyanov", Varna, BGR

**Keywords:** c-reactive protein, gram-negative bacteria, gram-positive bacteria, head and neck abscess, maxillofacial surgery, mean platelet volume, mean platelet volume-to-platelet count ratio, non-odontogenic abscesses, odontogenic abscesses, procalcitonin

## Abstract

Introduction

Head and neck abscesses, which can originate from odontogenic or non-odontogenic sources, pose significant diagnostic challenges due to their diverse bacterial etiologies. This study aims to investigate the impact of bacterial etiology on procalcitonin (PCT), C-reactive protein (CRP), and various hematological parameters, and to assess the diagnostic performance of mean platelet volume (MPV) in differentiating between Gram-negative bacteria (GNB) and Gram-positive bacteria (GPB) in adults with odontogenic and non-odontogenic head and neck abscesses.

Materials and methods

Our retrospective analysis of a prospective study comprised 80 patients: 50 individuals (56% men, average age 41.6±18.18 years) with odontogenic and 30 patients (66.7% men, average age 44.53±15.49 years) with non-odontogenic head and neck abscesses during the period from July 2021 to June 2022. White blood cell count (WBC); neutrophil (Neu) and lymphocyte (Ly) count; MPV, and platelet count (PLT) were derived from the results of complete blood count. MPV/PLT (MPI) was calculated by dividing MPV by PLT. CRP levels (mg/l) were quantified via immunoturbidimetric analysis utilizing latex-enhanced particles and PCT levels (ng/ml) by latex-enhanced immunoturbidimetric assay.

Results

In 25 (31.3%) of all 80 patients, no microorganisms were isolated (sterile cultures); in 28 (35%) resident microflora were isolated; in seven (8.8%) GNB were isolated; and in 17 (21.3%) GPB were isolated. CRP and Neu were significantly higher in patients with odontogenic abscesses compared to non-odontogenic ones. PLT and PCT were lower in patients with odontogenic abscesses vs those with non-odontogenic abscesses. Additionally, according to bacterial type, MPV, MPI and PCT were significantly higher in GPB compared to GNB. WBC, Neu and PLT were higher in patients with GNB vs GPB. Significant correlations were found between MPV and Ly, and between MPV and Neu, regardless of the abscess origin or etiological factor. MPI exhibited an area under the curve of the receiver operating characteristic (AUC-ROC)=0.776, MPV of 0.541, and PCT of 0.568 in distinguishing patients with GPB from GNB. A cut-off value of 0.029 was derived for MPI (70.6% sensitivity and 80% specificity).

Conclusions

This study highlights the impact of bacterial etiology on inflammatory and hematological markers in head and neck abscesses. Odontogenic abscesses showed higher CRP and Neu, indicating a stronger inflammatory response, while non-odontogenic abscesses had higher PLT, Ly, and PCT. MPI proved to be a more effective diagnostic marker (cut-off value of 0.029) than MPV or PCT for distinguishing between GPB and GNB, suggesting its valuable role in clinical practice for accurate and timely diagnosis.

## Introduction

An abscess, characterized by inflammation, is a localized accumulation of pus. Abscesses in the head and neck region are common yet potentially serious infections that can arise from a variety of causes, broadly categorized into odontogenic (originating from dental structures) and non-odontogenic (arising from other sources) [1,2]. Non-odontogenic abscesses usually arise from an infection that enters the soft tissues through various injuries to the oral mucosa or the skin in the head and neck region [3]. To identify an abscess as odontogenic, it is crucial to determine the presence of a causative tooth from which the inflammatory process begins, often referred to as the gateway of infection [1]. When the dental pulp chamber is exposed or opened, the root canals can become colonized by various strains of aerobic or anaerobic microorganisms [1]. These abscesses are not only a clinical challenge due to their potential for rapid progression and severe complications but also because of the diverse bacterial pathogens that can cause them. Understanding the bacterial etiology, particularly the distinction between Gram-positive and Gram-negative organisms, is crucial for effective diagnosis and treatment.

Odontogenic abscesses are typically caused by Gram-positive bacteria (GPB), such as *Streptococcus* and *Staphylococcus* species, which invade through dental caries, failed endodontic treatments, or other dental pathologies [1,4]. These infections can lead to localized pus formation, which, if not managed promptly, may spread to surrounding tissues or even disseminate systemically [1,4]. On the other hand, non-odontogenic abscesses, often associated with Gram-negative bacteria (GNB) such as *Escherichia coli* or *Klebsiella* species, usually result from trauma to the mucosal surfaces or skin in the head and neck region or from systemic infections that localize in these areas [5]. The inflammatory response to these bacterial infections can vary significantly depending on the type of pathogen involved. Biomarkers such as procalcitonin (PCT) and C-reactive protein (CRP) are critical in assessing the body's response to these infections. PCT is particularly valuable in identifying bacterial infections, often rising significantly in response to Gram-negative sepsis, whereas CRP levels generally increase in response to both GPB and GNB infections, reflecting the overall inflammatory burden [6].

In addition to these biomarkers, hematological parameters such as white blood cell count (WBC), mean platelet volume (MPV), and platelet count (PLT) provide further insight into the systemic effects of these infections. Platelets are crucial in various pathophysiological processes, including hemostasis, thrombosis, inflammation, and the body’s defense against microbial infections [7]. Upon activation, platelets engage with white blood cells (WBCs) such as lymphocytes, monocytes, and macrophages, contributing to anti-inflammatory responses [8]. Parameters related to platelets, like PLT and MPV, which are easily measured through routine blood tests, have gained recognition for their significance in managing inflammatory diseases. MPV serves as an indicator of platelet activation, and together with PLT and the MPV-to-PLT ratio (MPI), these markers are valuable in assessing the level of inflammatory activity and the effectiveness of treatment in infectious diseases [6]. The nature of the bacterial pathogen, whether GPB or GNB, can influence these parameters differently, affecting the severity and course of the infection [6].

This study aims to investigate the impact of bacterial etiology on PCT, CRP, and various hematological parameters, and to assess the diagnostic performance of MPV in differentiating between GNB and GPB in adults with odontogenic and non-odontogenic abscesses of the head and neck. By analyzing these factors, the research seeks to enhance our understanding of how different bacterial pathogens influence the clinical presentation and progression of abscesses in the head and neck region. The findings could lead to more tailored therapeutic strategies, improving outcomes for patients with these complex infections.

## Materials and methods

This is a retrospective reporting of a prospective study. The design is an observational comparison-group study. The same was conducted after approval by the Institutional Review Board of Medical University "Prof. Dr. Paraskev Stoyanov", Varna, Bulgaria (approval number: 101/2021). It included, studied and analyzed all 80 patients with odontogenic (n=50) and non-odontogenic (n=30) head and neck abscesses, who for a period of one year (from the beginning of July 2021 to the end of June 2022) were hospitalized in the Clinic of Maxillofacial Surgery at the University Hospital St. Marina, Varna, Bulgaria. In all of them, the diagnosis of abscess was confirmed initially during the physical examination by an oral or maxillofacial surgeon and then during the operative treatment in the volume of incision, lavage and drainage, during which a different amount of purulent exudate was evacuated.

Inclusion criteria were that all patients were 18 years of age or older and had been hospitalized and operated on for an odontogenic or non-odontogenic head or neck abscess.

The exclusion criteria included patients under the age of 18 and those with conditions or diseases that could artificially elevate levels of PCT, CRP, WBC count and PLT count. These conditions encompassed other infections (viral, bacterial, or fungal), parasitic infestations, recent major trauma or surgical procedures, fever, burns, oncological disorders, paraneoplastic syndromes, medications that promote cytokine production, cardiogenic shock, tissue hypoperfusion, bronchial asthma, and pulmonary pneumonia. Additionally, for parameters of complete blood count, the exclusion criteria also included immunosuppression, emotional stress, pregnancy, confirmed diagnoses of blood disorders (e.g., leukemia, thalassemia, immune thrombocytopenia, and multiple myeloma), and recent treatment, including anticoagulants, antiplatelet agents, hemostatic agents, granulocyte boosters, or clinical transfusion therapy (e.g., platelets, red blood cells, or plasma).

In the present study, we determined the mean values of key hematological parameters, including WBC, neutrophil (Neu) count, lymphocyte (Ly) count, MPV, PLT count and MPI. Blood samples were collected preoperatively from all participants following the diagnosis of head and neck abscesses. Whole blood was collected in vacutainers containing dipotassium ethylenediaminetetraacetic acid (K^2^EDTA) as an anticoagulant. Total WBC, Neu, Ly, and PLT counts were derived from routine complete blood count analyses performed using an automated 5-Diff hematology analyzer (ADVIA 2120, Siemens Healthineers, Erlangen, Germany). This advanced flow cytometry-based system employs light scatter, differential leukocytes lysis, and myeloperoxidase and oxazine 750 staining to yield a comprehensive blood cell profile. The results were reported in the following units: Nx10^9/L for WBC and PLT, Nx10^9/L or % for Neu and Ly count. MPI was calculated by dividing MPV by PLT and expressed as numerical value.

For CRP and PCT analyses, serum was separated by centrifugation at 2500 G for 15 minutes from blood collected in vacutainers equipped with gel separators. CRP levels were quantified via immunoturbidimetric analysis utilizing latex-enhanced particles on the Cobas® 6000 platform (Roche Diagnostics Corporation, Indianapolis, IN, USA), while PCT levels were measured using a latex-enhanced immunoturbidimetric assay on the ADVIA 1800 biochemical analyzer (Siemens Healthineers) in conjunction with a reagent kit provided by Diazyme Laboratories, Inc. (Poway, CA, USA). The results were reported in the following units: mg/L for CRP and ng/mL for PCT.

Statistical analyses were executed using the SPSS software package, version 19 (modified May 21, 2021; IBM Corp., Armonk, NY, USA) on a Windows 10.0 platform (Microsoft Corporation, Redmond, WA, USA). Numerical data were expressed as mean values ± standard deviation (SD). Descriptive statistics were initially employed to determine the central tendency and dispersion of the data. Pearson's correlation coefficient (r) was used to perform correlation analysis, evaluating the linear relationship between variables. Linear regression analysis, both univariate and multivariate, was utilized to examine relationships between independent and dependent variables. Factor analysis, coupled with independent-samples T-tests was conducted to identify significant differences between sample means. For the analysis of nominal data, non-parametric statistical methods such as the Chi-square test of independence were applied. Receiver operating characteristic (ROC) analysis and the calculation of the area under the curve (AUC) were employed to assess the sensitivity (Se) and specificity (Sp) of laboratory parameters, which facilitated the derivation of optimal cut-off values. A significance level (α) of 0.05 was maintained across all analyses, with the null hypothesis being rejected for p-values less than α (p<0.05).

## Results

The study included 80 patients divided into two groups: 50 patients with odontogenic abscesses (56% men) and 30 with non-odontogenic abscesses of the head and neck (66.7% men). Gender distribution was comparable between the groups (χ²=0.889, p=0.346). The mean age was 41.6±18.18 years in the odontogenic abscess group and 44.53±15.49 years in the non-odontogenic abscess group, with no significant difference (F=1.345, p=0.250).

In 25 (31.3%) of all patients, no microorganisms were isolated as the causative agents of the infection (sterile cultures). In 28 (35%) patients, representatives of the resident oral microflora were isolated, indicating a polyinfection caused by normal inhabitants of the human oral cavity. GNB were isolated in seven (8.8%) patients and GPB were isolated in 17 (21.3%) patients.

The predominant Gram-negative bacteria were *Escherichia coli* and *Klebsiella pneumoniae*, while the predominant Gram-positive bacteria were *Staphylococcus aureus*, *Staphylococcus anginosus*, and *Staphylococcus epidermidis*. The distribution of isolated bacteria was comparable between patients from the odontogenic abscess group and the non-odontogenic abscess group (χ²=2.931, p=0.569).

Data from the 80 patients are presented in Table 1. CRP and Neu were significantly higher in patients with odontogenic abscesses compared to non-odontogenic ones: 104.94±111.75 mg/l vs 36.85±56.29 mg/l, p=0.003 for CRP and 73.67±11.49% vs 68.11±12.45%, p=0.046 for Neu. PLT, PCT and Ly were lower in patients with odontogenic abscesses vs those with non-odontogenic abscesses: PLT (268.38±80.46x10^9/L vs 301.77±133.02x10^9/L, p=0.035); PCT (0.816±1.02 ng/ml vs 1.26±1.53 ng/ml, p=0.006); Ly (16.59±9.27% vs 22.37±10.57%; 1.64±0.70x10^9/L vs 2.13±0.91x10^9/L) (Table 1).

**Table 1:** Baseline characteristics: Mean values of laboratory parameters in clinical groups with head and neck abscesses. Independent-samples T-test was conducted to identify significant differences between sample means. n: number; SD: standard deviation; WBC: white blood cells; Neu: neutrophil count; Ly: lymphocyte count; MPV: mean platelet volume; PLT: platelet count; MPI: mean platelet volume-to-platelet count ratio; CRP: C-reactive protein; PCT: procalcitonin

Studied marker	Patients with odontogenic abscesses (n=50)		Patients with non-odontogenic abscesses (n=30)		Reference ranges	p-value
	Mean value	SD	Mean value	SD		
WBC (10^9/L)	11.17	4.429	10.33	3.19	3.79-10.33	non-significant
Neu (10^9/L)	8.45	4.34	7.24	3.23	1.78-7.00	non-significant
Neu (%)	73.67	11.49	68.11	12.45	39-77	0.046
Ly (%)	16.59	9.27	22.37	10.57	20-44	0.008
Ly (10^9/L)	1.64	0.70	2.13	0.91	1.07-3.12	0.013
MPV (fl)	8.63	1.21	8.57	1.36	6.0-10.0	non-significant
PLT (10^9/L)	268.38	80.46	301.77	133.02	140-440	0.035
MPI (numerical value)	0.038	0.028	0.034	0.015	/	non-significant
CRP (mg/l)	104.94	111.75	36.85	56.29	0-5	0.003
PCT (ng/ml)	0.816	1.02	1.26	1.53	0-0.05	0.006

When comparing the average levels of analyzed parameters against the causative agents of the infection, individuals with GPB exhibited higher values of Ly, MPV, MPI and PCT compared to those with GNB (p<0.05). Conversely, statistically significant differences favoring GNB were noted for WBC, Neu and PLT (p<0.05), as shown in Table 2. Although higher average values of CRP were reported in patients with GNB, the observed differences were not statistically significant (Table 2).

**Table 2:** Mean values of analyzed parameters of patients with isolated Gram-negative and Gram-positive bacteria. Independent-samples T-test was conducted to identify significant differences between sample means. n: number; SD: standard deviation; GNB: Gram-negative bacteria; GPB: Gram-positive bacteria; WBC: white blood cells; Neu: neutrophil; Ly: lymphocytes; MPV: mean platelet volume; PLT: platelet count; MPI: mean platelet volume-to-platelet count ratio; CRP: C-reactive protein; PCT: procalcitonin

Studied marker	Patients with GNB (n=7)		Patients with GPB (n=17)		Reference ranges	p-value
	Mean value	SD	Mean value	SD		
WBC (10^9/L)	12.29	1.68	9.81	3.21	3.79-10.33	0.035
Neu (10^9/L)	9.85	2.09	7.15	2.80	1.78-7.00	0.033
Neu (%)	79.50	6.23	71.71	11.82	39-77	0.048
Ly (10^9/L)	1.57	0.49	1.77	0.86	20-44	non-significant
Ly (%)	13.19	4.89	19.26	10.23	1.07-3.12	0.05
MPV (fl)	7.93	0.65	8.79	1.21	6.0-10.0	0.035
PLT (10^9/L)	447.29	144.14	255.47	58.31	140-440	<0.001
MPI (numerical value)	0.019	0.007	0.036	0.011	/	<0.001
CRP (mg/l)	124.12	106.20	84.26	129.36	0-5	non-significant
PCT (ng/ml)	0.93	1.51	1.15	1.26	0-0.05	<0.001

Additionally, in individuals with isolated GNB, significant correlations were observed between WBC and Neu (r=0.974, p<0.005); WBC and Ly (r=-0.760, p=0.047); Neu and Ly (r=-0.867, p=0.011), and CRP and PCT (r=0.862, p=0.013). In patients with isolated GPB, notable correlations included WBC and Neu (r=0.942, p<0.005); Neu and Ly (r=-0.648, p=0.005); WBC and CRP (r=0.413, p=0.099); Neu and CRP (r=0.519, p=0.033), and Ly and CRP (r=-0.566, p=0.018). Across all studied patients, significant associations were observed between WBC and CRP (r=0.549, p<0.05), WBC and Neu (r=0.583, p<0.05), WBC and Ly (r=-0.539, p<0.05), Neu and Ly (r=-0.270, p=0.016), Neu and CRP (r=0.594, p<0.05), and Ly and CRP (r=-0.343, p=0.02).

Furthermore, a significant positive correlation was found between MPV and Ly (r=0.294, p=0.008), and a negative association between MPV and Neu (r=-0.294, p=0.008) in all studied patients.

To establish the linear relationship between MPV as а dependent variable and Neu and Ly as independent variables, a multiple linear regression analysis was conducted. A statistically significant combination of the variables Ly % and Neu % was identified for predicting the value of MPV in adults with head and neck abscesses, F=3.795, p=0.027. The regression constant was 9.381, p=0.04. The equation found for the relationship between the variables was MPV(fl)=9.381+Ly(%)x0.154-Neu(%)x0.152.

**Figure 1:**
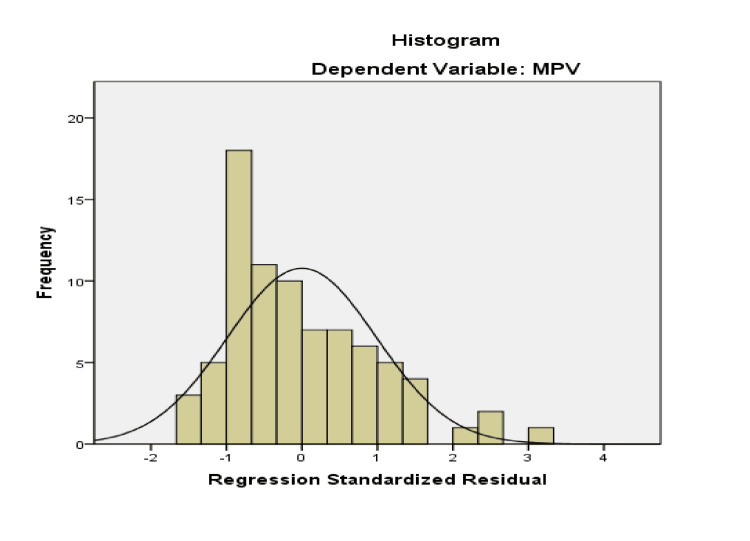
Histogram for normal distribution as a prerequisite for regression analysis MPV: mean platelet volume

**Figure 2:**
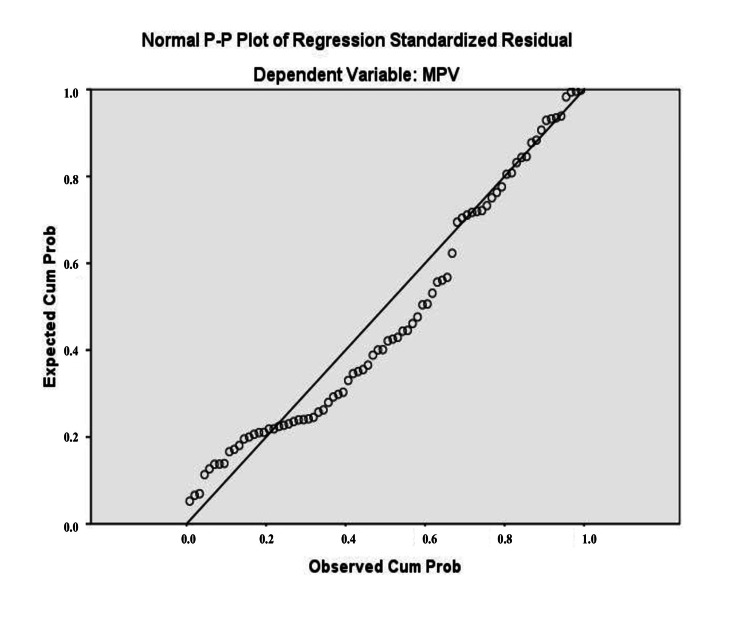
P-P Plot for normal distribution as a prerequisite for regression analysis MPV: mean platelet volume

**Figure 3:**
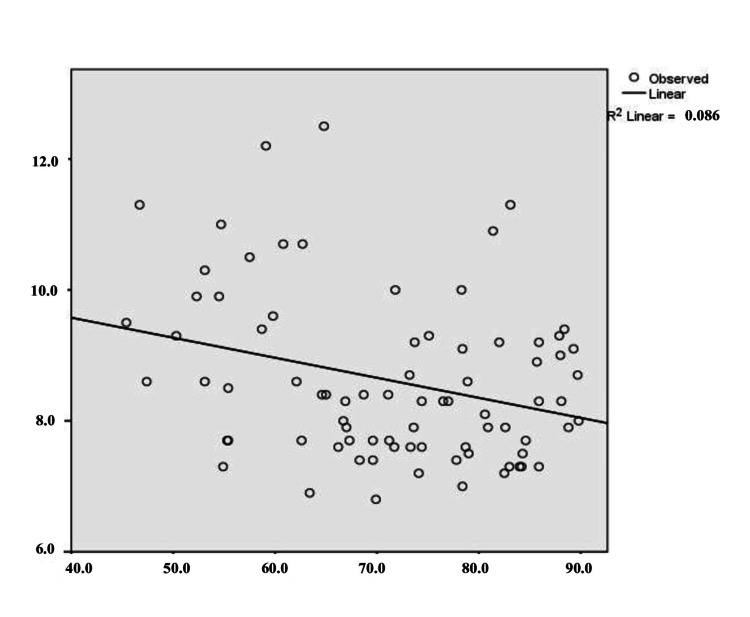
Regression curve: MPV (fl) as dependent variable; Neu (%) as predictor. On the x-axis, Neu values (%) are presented; on the y-axis, MPV values (fL) are presented. MPV: mean platelet volume, Neu: neutrophil

**Figure 4:**
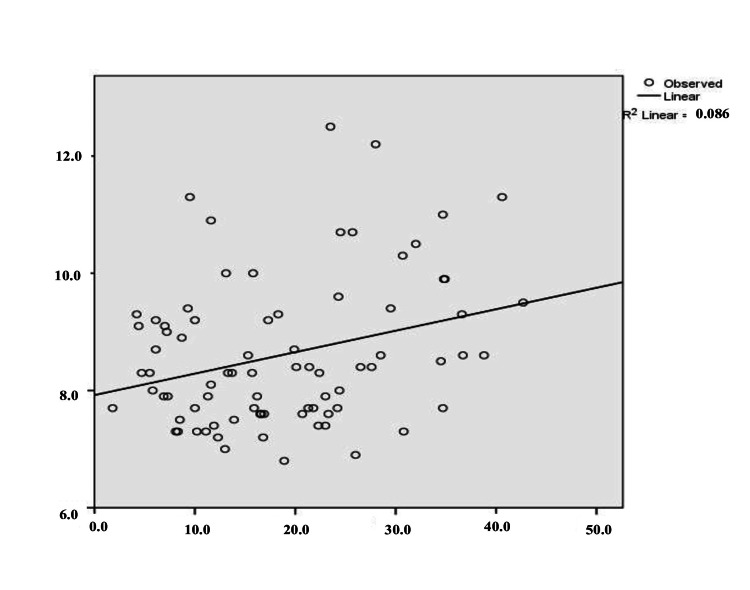
Regression curve: MPV (fl) as dependent variable; Ly (%) as predictor. On the x-axis, Ly values (%) are presented; on the y-axis, MPV values (fL) are presented. MPV: mean platelet volume, Ly: lymphocyte

The multiple linear regression equation, resulting from the analyzed data, predicts the dependent variable (MPV) based on the two independent variables, Ly and Neu. The relationship between the combined variables was moderate, with R=0.300. The adjusted R² was 0.09, indicating that 9% of the variation in MPV values is explained by the regression model. To verify homogeneity of variance, standardized residual plots were examined. Statistical tests and a histogram confirmed a normal Gaussian distribution of MPV in studied patients (Figure 1). The histogram illustrates the distribution of the variable in the form of bars. The P-P Plot shows the regression line closely follows the diagonal (Figure 2). Figure 3 and Figure 4 present the regression curves of analysis. The regression graphs illustrate how changes in the relative count of Neu and Ly influence MPV, specifically showing an inverse relationship between Neu and MPV, and a direct relationship between Ly and MPV.

To determine the cut-off values of MPV, MPI and PCT that distinguish groups with GPB from those with GNB, ROC curve analysis was applied. MPI exhibited an AUC-ROC of 0.776, p=0.018. MPV and PCT did not show good prognostic value, with an AUC-ROC for MPV=0.541, p=0.725, and an AUC-ROC for PCT=0.568, p=0.564.

**Figure 5:**
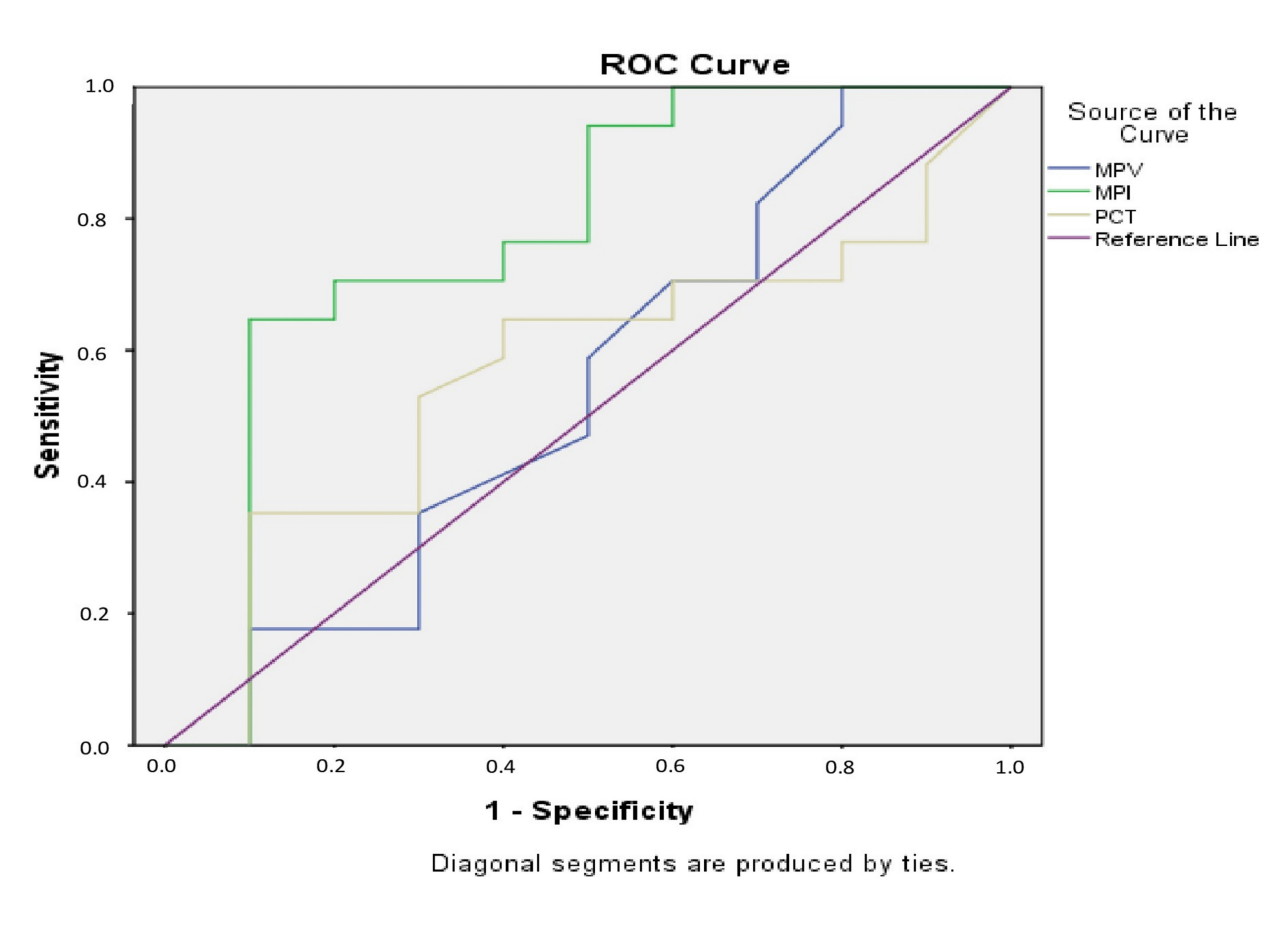
AUC-ROC curve to estimate a prognostic value of MPI, MPV and PCT in distinguishing patients with GPB from those with GNB AUC-ROC: area under the curve of the receiver operating characteristic; MPI: mean platelet volume-to-platelet count ratio; MPV: mean platelet volume; PCT: procalcitonin; GPB: Gram-positive bacteria; GNB: Gram-negative bacteria

A cut-off value of 0.029 was derived for MPI, yielding a sensitivity of 70.6% and specificity of 80%. The calculated likelihood ratios were LR(+)=3.5 and LR(-)=0.42 (Figure 5).

## Discussion

While the etiology and clinical progression of maxillofacial infections are well-documented, identifying factors unique to odontogenic and non-odontogenic infections requires a thorough investigation. These infections have distinct origins, influenced by various factors that modify their course, severity, and predisposition [9]. To address these complexities, this study offers a comprehensive analysis of head and neck infections, comparing odontogenic and non-odontogenic maxillofacial infections.

The results of our study on gender and age distribution of patients are similar to previous studies [9,10]. Considering the average age in the group of odontogenic and non-odontogenic abscesses, we confirm the highest frequency of these infections between the ages of 25-45, with a slight predominance in men, though statistically insignificant [9-11]. Therefore, we accept the thesis that similar risk factors influence the development of both odontogenic and non-odontogenic abscesses, with a tendency for higher incidence in young men aged between 25-45 years [9]. Some of the most commonly discussed predisposing risk factors are smoking and alcohol consumption. They cause damage to the mucous membrane of the mouth and throat, which is the first immune barrier against the spread of microorganisms. Disruption of the physiological immune balance can lead to the development of both odontogenic and non-odontogenic maxillofacial infections [9]. Another predisposing factor for maxillofacial infections is poor oral hygiene [9]. Traditionally attributed as an important risk factor for odontogenic infections, it can also significantly impair local immune defenses and promote the spread of potentially benign pharyngeal infections [1,9]. In the 25-45 age group, the consequences of neglecting oral hygiene, caries, and periodontitis may be most clearly marked, which, combined with limited access to a dentist, may result in a higher risk of maxillofacial infections. Finally, in this age group, pathologies related to the eruption of the lower third molars are most common, which are postulated as a frequent cause of severe odontogenic infections [9,12].

In 31.3% (n=25) of the 80 patients studied (50 with odontogenic abscesses and 30 with non-odontogenic abscesses), no microorganisms were isolated as the causative agents of the infection. Several factors could account for this, including improper sample collection, where the sample might contain only pus, which is often low in microorganisms, instead of also including tissue material adjacent to the abscess, which is typically rich in bacterial content. Other potential reasons include improper storage conditions, such as inappropriate environment, temperature, humidity, or exposure to sunlight, as well as an excessively long interval between sample collection and testing.

The second most common finding is the resident bacterial microflora, typically consisting of two or more bacterial species, predominantly Gram-positive (35%, n=28). This pattern is characteristic of neck abscesses from various origins, such as odontogenic, rhinogenic, dermatogenic, and post-traumatic [1,2]. The resident flora often protects the body against disease-causing organisms. However, under certain conditions, microorganisms that are part of a person’s resident flora may cause disease. Such conditions include the use of antibiotics, injury or surgery, and a weakened immune system (as occurs in people with HIV infection or cancer, those taking corticosteroids, and those undergoing cancer chemotherapy) [13].

In line with previous studies, we found that the primary microorganisms responsible for head and neck abscesses in patients over 18 years of age are GPB [2,9,13]. One of the most common species causing human diseases is *Staphylococcus epidermidis*, a finding that our study also supports [14]. These bacteria typically colonize the skin and mucous membranes without causing infections under normal conditions [14]. However, when the skin and mucosal epithelium are injured or in cases of immune system disorders, they can lead to purulent infections. They are less virulent than *Staphylococcus aureus*, a conclusion also confirmed by our study [2,14]. GNB are significantly less common in neck infections, with no specific reason identified for this occurrence [14]. Our findings indicate that GPB are three times more prevalent than GNB, a result that aligns with numerous studies in the literature [9,10,13,14].

In our analysis, several significant differences in the studied laboratory parameters were identified, though the distribution of isolated bacteria was similar between the groups. Additionally, when comparing the average levels of analyzed parameters relative to the causative agents of the infection, significant differences were observed across all variables, except for CRP and the absolute value of Ly.

In contrast to previous studies that found comparable results for CRP, we observed significantly higher values for this indicator, as well as a higher relative value of Neu in the group with odontogenic abscesses compared to those with non-odontogenic abscesses [6,9]. For WBC, we observed a similar trend, but it did not reach statistical significance. A higher level of CRP is linked to a more severe progression of maxillofacial infections [15]. The average CRP value we observed in the group with odontogenic abscesses was 104.94±111.75 mg/l, which is nearly three times higher than the value observed in the group with non-odontogenic abscesses, 36.85±56.29 mg/l, p=0.003. According to Pham Dang et al., patients with a CRP level exceeding 200 mg/l face a 27% risk of requiring multiple surgeries due to odontogenic infections [12]. Conversely, a CRP level below 50 mg/l, coupled with immunodepression, may predispose individuals to a more severe course of odontogenic infections [12]. Neu count and CRP levels are typically elevated, while Ly count is decreased in patients who develop abnormal inflammatory responses. According to Rosca et al. (2023), the association between CRP and Neu to Ly ratio was found to increase the risk of severe odontogenic infections by 7.28 times. The ROC analysis of CRP-Neu-to-Ly ratio yielded an AUC of 0.889, with high Se (79.6%) and high Sp (85.1%) for predicting severe odontogenic infections based on biomarkers measured at hospital admission (p<0.001) [15]. Kaminski et al. (2024) noted that these findings are attributed to the higher morbidity observed in patients with odontogenic infections compared to those with non-odontogenic infections [9].

Furthermore, we observed more severe leukocytosis, due to neutrophilia in patients with GNB, compared to those with GPB. In general, acute inflammation is characterized by an increase in Neu count, whereas chronic inflammation is typically associated with a rise in Ly levels. Inflammatory responses are characterized by the detection of damaged tissues by inflammatory cells, the selective accumulation of certain leukocyte subsets, and the subsequent elimination of harmful agents. In cases of systemic bacterial inflammation, a decrease in Ly count and an increase in Neu count are typically observed [16]. Lymphopenia is thought to result from the margination and redistribution of Ly within the lymphatic system, while neutrophilia is driven by the accumulation of Neu at the infection site, delayed apoptosis, and stem cell activation [16]. According to several authors, GNB are generally more harmful than GPB for several reasons [2,4,6,9]. They have a robust outer membrane that shields them from many antibiotics, making them more difficult to treat. Furthermore, when their cell walls are compromised, they release endotoxins that can intensify symptoms and provoke severe inflammatory reactions. This heightened resistance to antibiotics results in greater morbidity and mortality, thereby rendering GNB significantly more harmful than GPB [9]. In acccordance with Kaminski et al. (2024), we found no significant differences in CRP levels between GNB and GPB groups [9].

The positive correlation between CRP and WBC, as well as with the Neu count, has been extensively studied by various authors, whereas a negative correlation with the Ly count has also been observed [15-17]. Consequently, the results of our study confirm these associations, regardless of the abscess origin (odontogenic or non-odontogenic) or the etiological agent (GNB or GPB).

Our findings regarding the intergroup differences in average PCT levels were unexpected. We observed higher levels of this marker in individuals with non-odontogenic abscesses compared to those with odontogenic abscesses, as well as higher levels in GPB compared to GNB. This contrasts with several studies that have reported significantly higher PCT levels in patients with GNB compared to those with GPB [6,9]. The endotoxins on the cell walls of GNB may directly stimulate PCT production. Thomas-Ruddel et al. and Yu et al. noted that PCT serum concentrations can be influenced by the infection site, potentially limiting its diagnostic value for GNB [18,19]. A meta-analysis concluded that PCT is generally more effective than CRP for diagnosing GNB infections [20]. The discrepancy in PCT levels observed in our study may be due to differences in inflammatory responses and systemic inflammatory responses induced by these bacterial classes through distinct signaling pathways or possibly the limited size of the studied cohort. Recently, the role of the delta neutrophil index (DNI) in the diagnosis, follow-up and prediction of the outcome of the disease in inflammatory infections in the head and neck region has been discussed [21].

Platelets contribute to the pathogenesis of infectious diseases in addition to their primary role in hemostasis. Infections can lead to changes in platelet size, with an increase in MPV often seen during severe infections [6]. This increase in MPV is thought to result from the rapid release of platelets from the spleen, making MPV a useful inflammatory marker in the early stages of infection. Elevated MPV levels have been observed in conditions such as acute pyelonephritis, peritonsillar abscesses, acidic fluid infections, severe community-acquired pneumonia requiring hospitalization, and infective endocarditis [22,23]. The results of our study demonstrated that platelet parameters such as PLT, MPV, and MPI were significantly different between the GPB and GNB groups. Infections caused by GPB, such as *Staphylococcus*, *Streptococcus* and *Peptostreptococcus* spp, often lead to a localized inflammatory response [24]. This typically results in a smaller increase in MPV relative to the overall PLT count, potentially leading to a lower MPI. In contrast, infections caused by GNB, such as *Escherichia coli* and *Pseudomonas*, are associated with more widespread inflammation and septic responses. This systemic inflammation can cause a more significant increase in MPV due to the production of larger, more reactive platelets, which might result in a higher MPI [22]. The inverse relationship between the PLT count and MPV has previously been described [6]. We observed a similar trend in our study, but it did not reach statistical significance.

In contrast to Gao et al. (2021), we found that MPV and MPI in the GPB group were significantly higher than those in the GNB group, while the PLT count was significantly lower in the GPB group [6]. Although GNB trigger a more intense inflammatory response in the body, leading to higher concentrations of inflammatory factors than GPB, some studies have indicated that GPB are associated with greater platelet activation, consistent with our findings [25]. This difference can result in changes in platelet volume, aggregation, and volume distribution. However, the precise differences in hematological levels of these non-specific biomarkers still require further investigation at the molecular level to better understand the distinct pathogenic mechanisms involved. Furthermore, patient-specific factors and variations in the inflammatory response can affect MPV and PLT levels, making it essential to interpret these measurements in the context of the overall clinical picture [6].

Additionally, we observed a significant association between the MPV parameter and the relative counts of Ly and Neu across all studied patients, regardless of the abscess origin or etiological factor. Аccording to several authors, increased MPV is correlated with neutrophilia and lymphocytopenia, indicating a stronger inflammatory response in the body [16,22,23]. This correlation reflects the role of PLT as active participants in the immune response during infections, where larger, more reactive PLT are produced alongside an increase in Neu and a decrease in Ly. In summary, the association between increased MPV, neutrophilia, and lymphocytopenia underscores the dynamic nature of the immune response, highlighting how the body adapts to and manages inflammation and infection [16]. We were surprised to find an inverse correlation between MPV and both Neu and Ly in our investigation. Possible explanations for this phenomenon are as follows: during inflammatory or immune responses, both PLT and Ly can become activated. An increased MPV typically indicates heightened PLT activation and production, while elevated Ly counts reflect their role in the inflammatory process [8]. The body produces larger PLT in response to infection, and Ly, particularly T-cells, are mobilized to combat the pathogen. Conversely, conditions such as autoimmune disorders or chronic infections can result in both elevated MPV and increased Ly counts. The persistent inflammatory environment can drive the production of larger, more reactive PLT and enhance Ly proliferation [8].

Our study found that the diagnostic value of MPI was superior to either MPV or PCT in distinguishing GNB from GPB. MPI showed an AUC-ROC of 0.776 (p=0.018), highlighting its effectiveness as a diagnostic marker in this context. Additionally, we derived a cut-off value of 0.029 for MPI, which demonstrated a Se of 70.6% and Sp of 80%.

Limitations

As limitations of this article, it can be noted that it does not study the pediatric population, it covers only a one-year time interval during which the patients were studied, and it considers patients who were hospitalized in only one clinic of one hospital.

## Conclusions

In conclusion, our research provides valuable insights into the impact of bacterial etiology on inflammatory and hematological markers in head and neck abscesses. Inflammatory markers such as CRP and PCT showed significant variations based on bacterial etiology and abscess origin. Specifically, CRP and PCT levels were higher in patients with odontogenic abscesses compared to non-odontogenic ones, while MPV and PLT levels differed according to bacterial type. Notably, our study found that the diagnostic value of MPI was superior to either MPV or PCT in distinguishing GNB from GPB. MPV as a standalone marker does not have sufficient diagnostic accuracy. We derived a cut-off value of 0.029 for MPI, which demonstrated a Se of 70.6% and a Sp of 80%. These findings underscore the importance of precise bacterial identification and relevant laboratory tests for optimal treatment of these complex infections. Enhanced understanding of these relationships can lead to more accurate diagnoses and more effective therapeutic strategies, ultimately improving clinical outcomes for patients with head and neck abscesses.
